# Clinical trial design data for electrocardiogram artificial intelligence-guided screening for low ejection fraction (EAGLE)

**DOI:** 10.1016/j.dib.2019.104894

**Published:** 2019-11-27

**Authors:** Xiaoxi Yao, Rozalina G. McCoy, Paul A. Friedman, Nilay D. Shah, Barbara A. Barry, Emma M. Behnken, Jonathan W. Inselman, Zachi I. Attia, Peter A. Noseworthy

**Affiliations:** a Division of Health Care Policy and Research, Department of Health Sciences Research, Mayo Clinic, Rochester, MN, USA; b Robert D. and Patricia E. Kern Center for the Science of Health Care Delivery, Mayo Clinic, Rochester, MN, USA; c Department of Cardiovascular Medicine, Mayo Clinic, Rochester, MN, USA; d Division of Community Internal Medicine, Department of Medicine, Mayo Clinic, Rochester, MN, USA; e Knowledge and Evaluation Research Unit, Mayo Clinic, Rochester, MN, USA

**Keywords:** Electrocardiogram, Artificial intelligence, Clinical trial, Heart failure

## Abstract

The article details the materials that will be used in a clinical trial - ECG AI-Guided Screening for Low Ejection Fraction (EAGLE): Rationale and design of a pragmatic cluster randomized trial [1]. It includes a clinician-facing action recommendation report that will translate an artificial intelligence algorithm to routine practice and an alert when a positive screening result is found. This report was developed using a user-centered approach via an iterative process with input from multiple physician groups. Such data can be reused and adapted to translate other artificial intelligence algorithms. This article also includes data collection forms we developed for the clinical trial aiming to evaluate the artificial intelligence algorithm. Such materials can be adapted for other clinical trials.

**Table:** Specifications Table

Subject	Cardiology and Cardiovascular Medicine
Specific subject area	Heart failure
Type of data	Figure
How data were acquired	The data were obtained via the discussion within the investigative team and interviews with clinicians from a variety of specialties. The data were created by the investigators using simple software like Word and pdf.
Data format	Raw
Parameters for data collection	Data were collected via discussion and interviewers with multiple stakeholders including cardiologists, health services researchers, primary care clinicians, emergency room physicians, anesthesiologists, designers, statisticians, study coordinators, etc.
Description of data collection	Data were collected via discussion and interviews.
Data source location	Mayo Clinic Minnesota and Wisconsin United States
Data accessibility	With the article
Related research article	same author list as this paper ECG AI-Guided Screening for Low Ejection Fraction (EAGLE): Rationale and design of a pragmatic cluster randomized trial American Heart Journal 10.1016/j.ahj.2019.10.007

**Table:** 

**Value of the Data** • These data provide an example of how an artificial intelligence algorithm can be translated to practice and how to design a clinical trial to evaluate the value of the algorithm in routine clinical practice. • Clinicians and researchers who are working on translating artificial intelligence algorithms to routine practice and who are designing clinical trials. • Clinicians and researchers can use these materials as a start point and adapt to their own projects.

## 1 Data

**Fig. 1:**
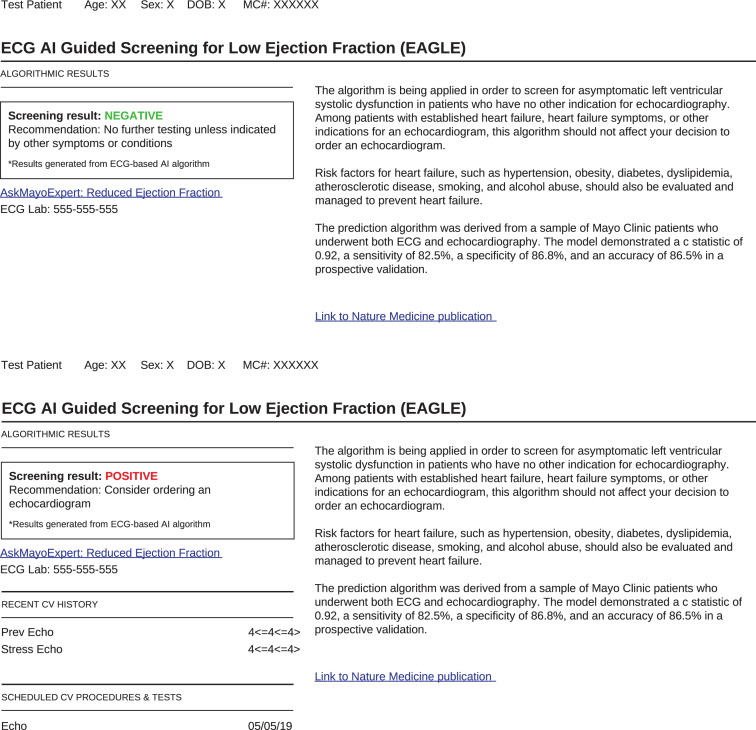
Sample clinician-facing report for ECG AI guided screening for low ejection fraction (EAGLE).

**Fig. 2:**
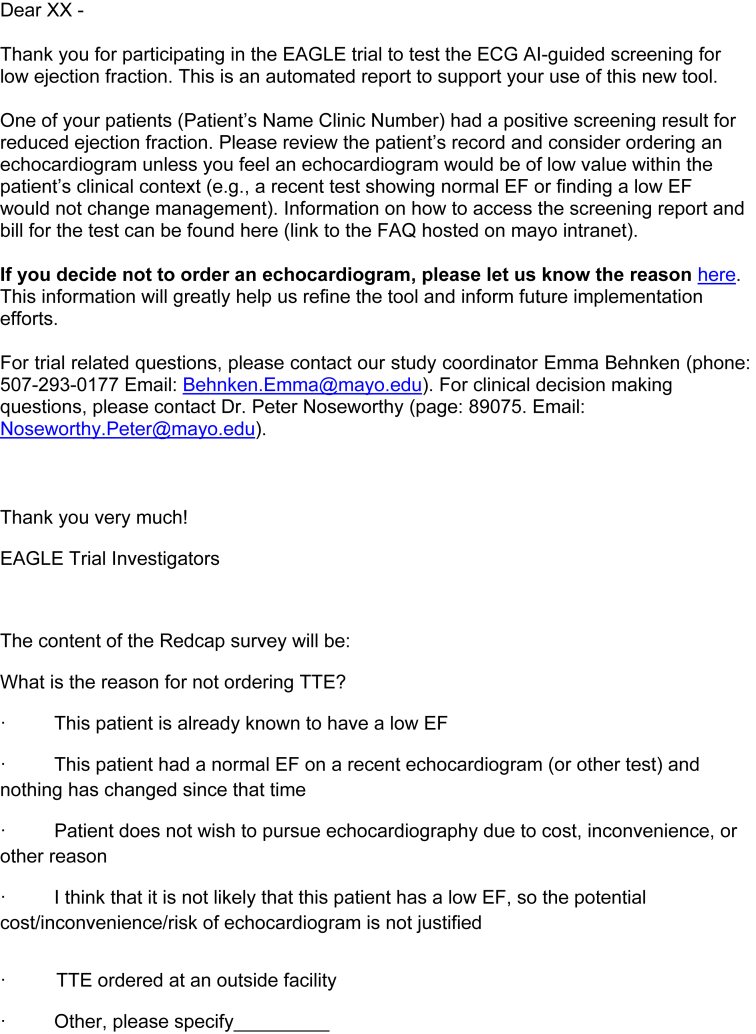
Sample email alert to clinicians when a positive screening result is detected.

**Fig. 3:**
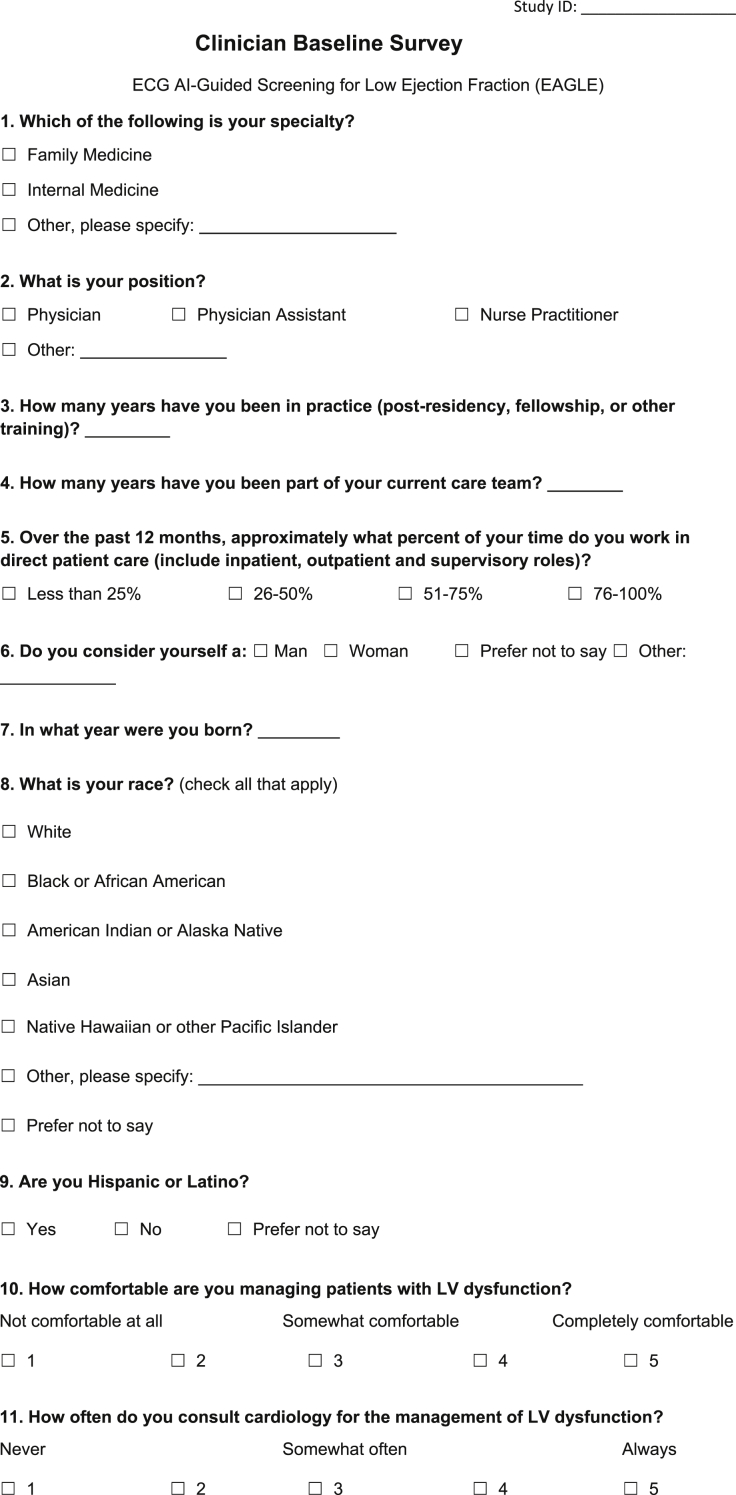
Clinician baseline survey.

**Fig. 4:**
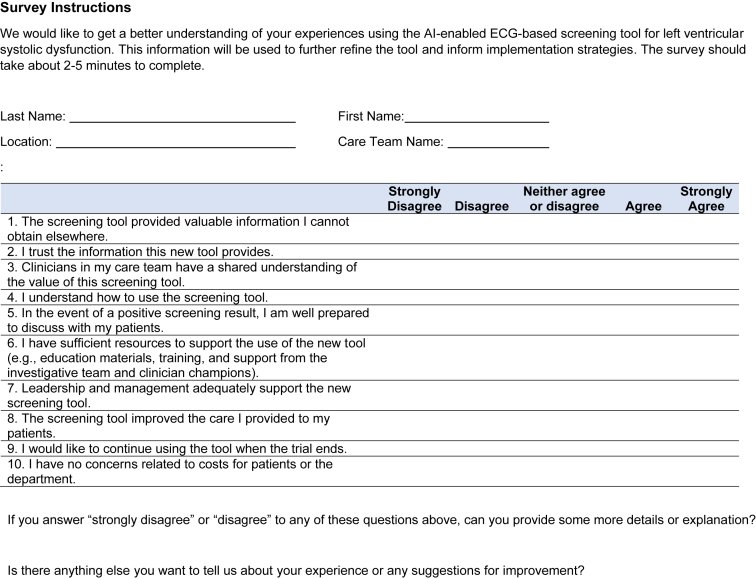
Clinician end-of-study survey.

Fig. 1 includes a clinician-facing action recommendation report with two versions – one for a negative result which requires no action, and the other for a positive result, which suggests ordering an echocardiogram. Fig. 2 is a sample email alert to clinicians when a positive screening result is detected. Fig. 3 is the baseline survey that will be administered to clinicians at the time of enrolment. Fig. 4 is the end-of-study survey that will be administered to clinicians in the intervention group at the end of the trial [1].

## 2 Experimental design, materials, and methods

The clinician-facing action recommendation report was developed over a period of four months (December 2018–March 2019). A multi-disciplinary team developed a prototype of the report using a user-centered iterative approach. The principal investigators of the project (a health services researcher and a cardiologist) drafted an initial prototype. The investigative team then identified major groups of clinicians who frequently order ECG (i.e., those in primary care, cardiology, emergency medicine, and anesthesiology) and introduced the tool to the leadership of these departments during face-to-face meetings. At these stakeholder meetings, the investigative team got a better understanding of their needs and solicited feedback on the new tool and the design of the report. The investigative team also asked the department leaders to suggest 3–5 practicing clinicians in each department to participate in the subsequent testing and refinement of the prototype. Two designers worked with practicing clinicians to conduct interviews and workflow observations. A series of prototypes were developed, tested, and revised based on these clinicians' feedback. The investigative team met regularly to discuss the iterations of the prototype and the clinicians' feedback. The prototype was also tested with five clinicians using real patient data and was then finalized based on the feedback. Other trial materials were developed by the multi-disciplinary team including physicians from cardiology and primary care, health services researchers, statisticians, and a study coordinator.
